# Clinical effect of different concentrations of ropivacaine in the labor analgesia of dural puncture epidural technique for obese puerperae

**DOI:** 10.1186/s13741-024-00363-1

**Published:** 2024-02-15

**Authors:** Liping Shi, Difei Zhang, Pengfei Ye, Weihua Peng, Yan Yin, Ye Zhang

**Affiliations:** Department of Anesthesiology, Taihe County People’s Hospital, Fuyang, 236600 Anhui China

**Keywords:** Ropivacaine, Labor analgesia, Dural puncture epidural technique, Obese puerperae, Clinical effect

## Abstract

**Background:**

This study was performed to analyze the clinical effect of different concentrations of ropivacaine in the labor analgesia of the dural puncture epidural (DPE) technique for obese puerperae.

**Methods:**

One hundred and fifty first-term obese women who received vaginal delivery and required labor analgesia in our hospital were selected prospectively for this study, and divided into groups A, B, and C. The three groups of puerpera were given epidurals with different concentrations of ropivacaine (0.075%, 0.10%, and 0.125%) with sufentanil (0.5 μg/ml) for the labor analgesia regimen. The visual analog scale (VAS), Ramsay scale, and Bromage scale of puerperae before analgesia and at different time points after anesthesia, and analgesic onset time, analgesia time, first PCEA time, PCEA pressing time, ropivacaine consumption, labor time, maternal blood pressure and heart rate, maternal adverse reactions, blood gas analysis in the neonatal umbilical artery, and Apgar score were observed.

**Results:**

The analgesia onset time, PCEA pressing time, and ropivacaine consumption in group C were lower and the analgesia time and the first PCEA time were longer than those in groups A and B. At T1-T3 and T5, VAS scores of group A were higher than those in groups B and C, Ramsay score of group A was lower than that of groups B and C at T2–T3, and Bromage score of group C at any time point was higher than other two groups. The time of the second stage of labor in groups B and C was longer than that in group A, which in group C was longer than that in group B. Compared with groups A and C, the blood pressure and heart rate of puerperae in group B were closer to normal values. Three different concentrations of ropivacaine had no significant effect on the umbilical artery blood gas analysis indices and Apgar scores at 1st minute and 5th minute in neonates. The incidence of maternal adverse reactions in group C was lower than those in groups A and B.

**Conclusion:**

0.1% ropivacaine combined with 0.5 μg/ml sufentanil through DPE technique has good analgesic efficacy and few adverse effects in obese puerperae.

**Supplementary Information:**

The online version contains supplementary material available at 10.1186/s13741-024-00363-1.

## Introduction

Pain control and the stress related to childbirth are some of the most essential issues in the health care system (Amiri et al. 2019). Currently, the gold standard for pain control in delivery patients is a neuraxial blockade, which consists of an epidural, spinal, or combined spinal-epidural method (Koyyalamudi et al. 2016). Other drugs related to the neuraxial block include nitrous oxide, opioids, non-opioids, distraction therapy, and patient-controlled epidural analgesia (PCEA) (Miyakoshi et al. 2013; Likis et al. 2014). Labor analgesia effectively diminishes the parturient fear, gets full rest during the whole labor process, and helps to enhance the fetal oxygenation function, so as to enable the parturient to build self-confidence (Mao et al. 2022). Epidural analgesia is considered the standard for labor analgesia with sizeable analgesic effects, which yields higher satisfaction among pregnant women in comparison to other analgesic methods (Ren et al. 2021). The dural puncture epidural (DPE) technique is able to improve the analgesia quality by confirming midline placement and enhancing the intrathecal translocation of epidural drugs (Tan et al. 2022). With the elevated requirements for labor comfort, the requirement for anesthesia accuracy is increasingly high (Xu et al. 2021).

With the improvement of maternal demands for labor analgesia, epidural analgesia is widely applied in labor, thus relieving severe pain effectively during delivery (Chen et al. 2021). Local anesthetic medications, together with opioids, are presently regarded as the ideal regimen for epidural labor analgesia (Cai et al. 2020). Ropivacaine is a novel long-acting amide local anesthetic characterized by low toxicity to the central nervous and cardiovascular system (Wen et al. 2021). Sufentanil has been used in many articles as a local anesthetic adjuvant for epidural labor analgesia (Zhang T et al. 2019; Xiang et al. 2021), and the combination of which with ropivacaine is used in the present work. Wang et al. have stated that the combination of ropivacaine with sufentanil provides an effective analgesic impact for labor analgesia, without significant adverse effects and delayed labor progress (Wang F et al. 2009). Nowadays, PCEA is mainly used for intraspinal block delivery analgesia with low concentrations of local anesthetics. This technique is more individualized and can meet the analgesic demands of different puerpera and reduce the dosage (Zhang T et al. 2019). Moreover, a recent study has demonstrated that sufentanil PCEA with ropivacaine is safe for parturients and fetuses by reducing maternal pain and ensuring delivery comfort, which does not prolong the labor process and impact the delivery process and fetal safety (Mao et al. 2022). Here in this research, we aimed to analyze the clinical effect of different concentrations of ropivacaine in the labor analgesia of the DPE technique for obese puerperae.

## Materials and methods

### Ethical approval

This study got approval from the ethics committee of the Taihe Hospital of Wannan Medical College (approval number: 20180319), and the mothers and their families signed the informed consent.

### Participants

Using a prospective study approach, a total of 150 obese primiparas who were admitted to the Taihe Hospital of Wannan Medical College and required labor analgesia from June 2018 to March 2021 were selected for our study. The general conditions of the puerperae are shown in Table 1.

**Table 1:** Comparison of the maternal general conditions

General information	Group A (*n* = 50)	Group B (*n* = 50)	Group C (*n* = 50)	*P* value
Age (year)	29.20 ± 2.79	30.14 ± 3.23	29.50 ± 3.04	0.289
BMI (kg/m^2^)	31.96 ± 1.73	32.12 ± 1.77	32.28 ± 1.86	0.549
Gestational week (week)	39.46 ± 0.97	39.54 ± 1.03	39.28 ± 1.18	0.459
ASA I/II class	21/29	27/23	20/30	0.315
Hypertension [case (%)]	12 (24.00%)	10 (20.00%)	14 (28.00%)	0.645
Diabetes [case (%)]	19 (38.00%)	20 (40.00%)	17 (34.00%)	0.819

*BMI* body mass index, *ASA* American Society of Anaesthesiologists, *Group A* 0.075% ropivacaine + 0.5 μg/ml sufentanil; *Group B* 0.10% ropivacaine + 0.5 μg/ml sufentanil; *Group C* 0.125% ropivacaine + 0.5 μg/ml sufentanil

Inclusion criteria were primiparous women aged 21–35 years old with American Society of Anaesthesiologists (ASA) class I-II, gestational age of 37–42 weeks, singleton pregnancy, fetus in a longitudinal position, body mass index (BMI) of 30–40 kg/m^2^, and met the conditions for vaginal delivery according to the obstetrician’s judgment. We excluded primiparous women with a contraindication to epidural analgesia, an allergy to ropivacaine or sufentanil, and combined with severe preeclampsia or malignancy. Withdrawal criteria were primiparous women who failed to perform epidural anesthesia, with a visual analog scale (VAS) less than 3 after loading dose, or converted to laparotomy.

### Grouping and analgesia

The primiparous enrolled in this research were randomly (computer-generated randomization and concealment via sealed opaque envelope technique) assigned to three groups (*n* = 50 in each group). When the puerpera had regular contractions, the veins of the upper extremity were open when the uterine orifice was 3 cm, and 500 mL of lactate Ringer’s solution (speed 5 mL/kg/h, Sichuan Kelun Pharmaceutical Co., Ltd., Sichuan, China) was infused. The maternal vital signs were routinely monitored, and the fetal heart rate was continuously monitored. The epidural analgesia was performed by 2 anesthesiologists with more than 10 years of clinical experience in labor analgesia, and the L3–L4 intervertebral space was selected. Epidural puncture was performed in strict accordance with the procedure of spinal canal puncture, and intra-needle needle technique, that is, a 16-gauge epidural puncture needle was used for puncture. After reaching the epidural space, a 27-gauge spinal needle was utilized to puncture the dura to the subarachnoid space through the epidural needle. When the cerebrospinal fluid refluxed, the spinal needle was withdrawn, and an epidural catheter was placed 4 cm upward. Next, a sterile syringe was connected and withdrawn gently until no blood and cerebrospinal fluid flowed out, followed by an injection of 3 mL of 1% lidocaine (Shandong Hualu Manufacturing Co., Ltd., Shandong, China; specification: 5 mL: 0.1 g) through an epidural catheter, followed by an observation for 5 min without any abnormality. Subsequently, the possibility of placing an epidural catheter into a blood vessel or subarachnoid space was excluded, and a patient-controlled epidural analgesia (PCEA) was connected. Group A (50 cases): 0.075% ropivacaine (AstraZeneca UK Limited.; specification: 10 ml: 100 mg) + 0.5 μg/ml sufentanil (Yichang Humanwell Pharmaceutical Co., Ltd., Hubei, China; specification: 1 mL: 50 μg) (diluted 7.5 ml of ropivacaine hydrochloride injection, 50 μg of sufentanil injection together with 0.9% sodium chloride injection to 100 ml and incorporated into the pump); group B (50 cases): 0.10% ropivacaine + 0.5 μg/ml sufentanil (diluted 10 ml of ropivacaine hydrochloride injection, 50 μg of sufentanil injection together with 0.9% sodium chloride injection to 100 ml and incorporated into the pump); group C (50 cases): 0.125% ropivacaine + 0.5 μg/ml sufentanil (diluted 12.5 ml of ropivacaine hydrochloride injection, 50 μg of sufentanil injection together with 0.9% sodium chloride injection to 100 ml and incorporated into the pump). The first dose was 10 mL, the background dose was 6 ml/h-8 ml/h, the single dose was 3 mL, and the locking time was 15 min. The mother or her family was instructed to use the analgesic pump correctly, and the PCEA button was pressed when the mother felt pain, and the analgesia would last until the end of labor (Wang J et al. 2021; Ran et al. 2022).

### Labor analgesia-related indicators

Starting from the injection of anesthetics, the analgesic onset time (the time when the sensory block level reached T10 level), the analgesia time (the time from the onset of analgesia to the occurrence of pain after childbirth), first PCEA time, PCEA pressing times, ropivacaine consumption, and other parameters were recorded.

### Visual analog scale (VAS)

The VAS was implemented to evaluate the pain of the puerpera. A 10-cm straight line was drawn on the paper, and the grid was divided based on millimeters. One end was painless and the other end was extremely painful. After visual inspection of the puerpera, a pen was utilized to draw a point on a straight line that matches its pain intensity according to her own situation and observe the distance of the point on the straight line. This operation was repeated 3 times to obtain the average value. The VAS scores of three groups of puerperae were observed and recorded before analgesia (T0), 10 min after analgesia (T1), 30 min after analgesia (T2), 1 h after analgesia (T3), 2 h after analgesia (T4), the cervix full-dilated time (T5), and the time of fetal delivery (T6). The score graded from 0 to 10 corresponded to painless and extremely painful (Sah et al. 2007).

### Ramsay scale

The Ramsay scale of three groups of puerperae was observed and recorded before analgesia (T0), 10 min after analgesia (T1), 30 min after analgesia (T2), 1 h after analgesia (T3), 2 h after analgesia (T4), the cervix full-dilated time (T5), and the time of fetal delivery (T6). Ramsay scale included 6 dimensions: 1 point, anxiety, excitement, or restlessness; 2 points, co-operation, and tranquility; 3 points, only responding to commands; 4 points, mild response to a loud auditory stimulus or light glabellar tap; 5 points, quick response to a loud auditory stimulus or light glabellar tap; 6 points, no response. This operation was repeated 3 times for obtaining the average value (Wu et al. 2021).

### Bromage scale

The Bromage scale of three groups of puerperae was observed and recorded 10 min after analgesia (T1), 30 min after analgesia (T2), 1 h after analgesia (T3), 2 h after analgesia (T4), the cervix full-dilated time (T5), and the time of fetal delivery (T6). The Bromage scale included 3 dimensions: 0 point, no motor block (full flexion of hips, knees, and ankles); 1 point, unable to flex the hips; 2 points, unable to flex the hips and knees; 3 points, complete block of the lower extremity. This operation was repeated 3 times for obtaining the average value (Zhong et al. 2020).

### Analysis of maternal delivery and neonatal status

The duration of the first, second, and third stages of labor was recorded for the three groups of puerperae. During the analgesia process, the three groups of puerperae were given routine oxygen inhalation, and their changes in blood pressure and heart rate were monitored in real time.

After delivery of the fetus, 2 mL of umbilical arterial blood was collected, and the partial pressure of carbon dioxide (PCO_2_), pH, and oxygen partial pressure (PO_2_) were analyzed using a blood gas analyzer (ABL80 FLEX; Radiometer Medical ApS, USA).

The Apgar score was performed on the newborns at 1st minute and 5th minute after delivery, respectively. The Apgar scoring standard was based on the five signs of neonatal skin color, heart rate, respiration, muscle tone, and motor reflex. A fetus whose whole body was pink was scored as 2 points, the extremities of the hands and feet were blue-purple, 1 point, and the whole body was blue-purple, 0 point; heartbeat > 100 beats/min was scored as 2 points, < 100 beats/min, 1 point, and no heart sound, 0 point; normal breathing was scored as 2 points, irregular breathing, 1 point, and no breathing, 0 point; the normal muscular tension was scored as 2 points, hypertonia or hypotonia, 1 point, and muscle relaxation, 0 point; when bouncing the sole of the fetus, loud crying was scored for 2 points, sobbing in a low voice or frowning, 1 point, and no response, 0 point (Patel et al. 2014).

### Adverse reactions

The occurrence of adverse reactions such as hypotension, nausea and vomiting, skin itching, fever, and urinary retention during labor analgesia in each group of puerperae were observed and recorded respectively. Incidence of adverse reactions (%) was calculated with (number of mothers with adverse reactions/total number of mothers) × 1 00%.

### Statistical analysis

All data were analyzed using SPSS 21.0 statistical software (IBM SPSS Statistics, Chicago, IL, USA), and measurement data were expressed as mean ± standard deviation. The t-test was used for comparison between two groups, and one-way ANOVA was used for comparison among multiple groups. Enumeration data were expressed as percentages or rates, and Fisher’s exact test or *χ*2 test was used for comparison between groups. *P* less than 0.05 was considered a statistically significant difference.

## Results

### Maternal general conditions

No significant differences were witnessed in the general data of maternal age, BMI, gestational week, ASA grade, hypertension, and diabetes (all *P* > 0.05; Table 1).

### Labor analgesia-related indices

As evidenced by the results in Table 2, the analgesic onset time, the PCEA pressing time, and the ropivacaine consumption were lower in group C (0.125% ropivacaine + 0.5 μg/ml sufentanil) than those in group A (0.075% ropivacaine + 0.5 μg/ml sufentanil) and group B (0.10% ropivacaine + 0.5 μg/ml sufentanil). These labor analgesia-related indices in group B (0.10% ropivacaine + 0.5 μg/ml sufentanil) were also lower than those in group A (0.075% ropivacaine + 0.5 μg/ml sufentanil) (all *P* < 0.05). Compared with group A (0.075% ropivacaine + 0.5 μg/ml sufentanil), the analgesia time and the first PCEA time were prolonged in group B (0.10% ropivacaine + 0.5 μg/ml sufentanil), and these indices were higher in group C (0.125% ropivacaine + 0.5 μg/ml sufentanil) than those in the other two groups (all *P* < 0.05).

**Table 2:** Comparison of labor analgesia-related indices among puerperae in each group

Efficacy	Group A (*n* = 50)	Group B (*n* = 50)	Group C (*n* = 50)
Analgesic onset time (min)	12.21 ± 2.35	9.52 ± 2.98#	6.17 ± 1.84#&
Analgesia time (min)	352.04 ± 89.25	386.30 ± 75.61#	424.64 ± 102.36#&
First PCEA time (min)	75.2 ± 24.7	106.52 ± 27.48#	130.64 ± 30.32#&
PCEA pressing times	4.36 ± 2.13	3.04 ± 0.97#	2.36 ± 0.98#&
Ropivacaine consumption (mg)	94.80 ± 12.95	85.56 ± 15.57#	70.64 ± 13.71#&

#*P* < 0.05 vs. Group A; ^&^ *P* < 0.05 vs. Group B. *PCEA* patient-controlled extracellular analgesia. *Group A* 0.075% ropivacaine + 0.5 μg/ml sufentanil; *Group B*, 0.10% ropivacaine + 0.5 μg/ml sufentanil; *Group C*, 0.125% ropivacaine + 0.5 μg/ml sufentanil

*VAS scores*

Before labor analgesia, no difference was witnessed in the VAS scores of puerperae in the three groups (*P* > 0.05), while at 10 min after analgesia (T1) to the time of fetal delivery (T6), the VAS scores of puerperae in the three groups were less than those before analgesia (*P* < 0.05). In contrast to group A (0.075% ropivacaine + 0.5 μg/ml sufentanil), the VAS scores at 10 min after analgesia (T1), 30 min after analgesia (T2), 1 h after analgesia (T3), and the cervix full-dilated time (T5) was decreased in group B (0.10% ropivacaine + 0.5 μg/ml sufentanil) and group C (0.125% ropivacaine + 0.5 μg/ml sufentanil) (all *P* < 0.05). The VAS scores at 10 min after analgesia (T1), and 30 min after analgesia (T2) were reduced in group C (0.125% ropivacaine + 0.5 μg/ml sufentanil) versus those in group B (0.10% ropivacaine + 0.5 μg/ml sufentanil) (*P* < 0.05) (Table 3).

**Table 3:** Comparison of VAS scores of three groups of puerperae at different time points ($\overline{x }$** ±** SD)

VAS scores	Group A (*n* = 50)	Group B (*n* = 50)	Group C (*n* = 50)
T0	8.60 ± 1.11	8.44 ± 1.07	8.54 ± 1.33
T1	4.98 ± 1.04*	4.60 ± 0.81*#	4.24 ± 0.82*#&
T2	2.42 ± 0.81*	2.10 ± 0.76*#	1.78 ± 0.71*#&
T3	2.10 ± 0.68*	1.84 ± 0.62*#	1.62 ± 0.49*#
T4	1.82 ± 0.77*	1.80 ± 0.95*	1.70 ± 0.95*
T5	3.78 ± 0.84*	3.40 ± 0.93*#	3.16 ± 0.79*#
T6	4.26 ± 1.32*	4.28 ± 1.16*	4.10 ± 1.16*

*P < 0.05 vs. before analgesia; #* P* < 0.05 vs. group A at the same time point; &* P* < 0.05 vs. Group B at the same time point. *Group A*, 0.075% ropivacaine + 0.5 μg/ml sufentanil; *Group B*, 0.10% ropivacaine + 0.5 μg/ml sufentanil; *Group C*, 0.125% ropivacaine + 0.5 μg/ml sufentanil. *T0* before analgesia; *T1* 10 min after analgesia, *T2* 30 min after analgesia, *T3* 1 h after analgesia, *T4* 2 h after analgesia, *T5* the cervix full-dilated time, *T6* the time of fetal delivery

### Ramsay scores

Before labor analgesia, no difference was witnessed in the Ramsay scores of puerperae in the three groups (*P* > 0.05), while at 10 min after analgesia (T1) to the time of fetal delivery (T6), the Ramsay scores of puerperae in the three groups were more than those before analgesia (*P* < 0.05). In contrast to group A (0.075% ropivacaine + 0.5 μg/ml sufentanil), the Ramsay scores at 30 min after analgesia (T2), and 1 h after analgesia (T3) were elevated in group B (0.10% ropivacaine + 0.5 μg/ml sufentanil) and group C (0.125% ropivacaine + 0.5 μg/ml sufentanil) (all *P* < 0.05). The VAS score at 30 min after analgesia (T2) was elevated in group C (0.125% ropivacaine + 0.5 μg/ml sufentanil) versus those in group B (0.10% ropivacaine + 0.5 μg/ml sufentanil) (*P* < 0.05) (Table 4).

**Table 4:** Comparison of Ramsay scores of three groups of puerperae at different time points ($\overline{x }$** ±** SD)

Ramsay scores	Group A (*n* = 50)	Group B (*n* = 50)	Group C (*n* = 50)
T0	1.00 ± 0.00	1.02 ± 0.14	1.00 ± 0.00
T1	1.50 ± 0.51*	1.52 ± 0.50*	1.58 ± 0.50*
T2	1.92 ± 0.40*	2.14 ± 0.45*#	2.38 ± 0.57*#&
T3	2.04 ± 0.45*	2.26 ± 0.56*#	2.42 ± 0.57*#
T4	2.28 ± 0.50*	2.32 ± 0.59*	2.44 ± 0.58*
T5	2.06 ± 0.59*	2.08 ± 0.40*	2.24 ± 0.56*
T6	1.60 ± 0.49*	1.72 ± 0.45*	1.76 ± 0.66*

* P < 0.05 vs. before analgesia; #* P* < 0.05 vs. Group A at the same time point; &* P* < 0.05 vs. Group B at the same time point. *Group A*, 0.075% ropivacaine + 0.5 μg/ml sufentanil; *Group B*, 0.10% ropivacaine + 0.5 μg/ml sufentanil; *Group C*, 0.125% ropivacaine + 0.5 μg/ml sufentanil. *T0* before analgesia, *T1* 10 min after analgesia, *T2* 30 min after analgesia, *T3* 1 h after analgesia, *T4* 2 h after analgesia, *T5* the cervix full-dilated time, *T6* the time of fetal delivery

### Bromage scores

All three groups of puerperae had a Bromage score of 0–2 points during childbirth, without a Bromage score of more than 2 points. In comparison to group A (0.075% ropivacaine + 0.5 μg/ml sufentanil), the Bromage scores of puerperae in group B (0.10% ropivacaine + 0.5 μg/ml sufentanil) were increased at 1 h after analgesia (T3) and 2 h after analgesia (T4), while the Bromage scores in group C (0.125% ropivacaine + 0.5 μg/ml sufentanil) were greater than those in the other two groups at any time point (all *P* < 0.05) (Table 5).

**Table 5:** Comparison of Bromage scores of three groups of puerperae at different time points ($\overline{x }$** ±** SD)

Bromage scores	Group A (*n* = 50)	Group B (*n* = 50)	Group C (*n* = 50)
T1	0.58 ± 0.50	0.62 ± 0.49	0.88 ± 0.52#&
T2	0.72 ± 0.45	0.78 ± 0.46	1.20 ± 0.61#&
T3	0.92 ± 0.63	1.18 ± 0.66 #	1.54 ± 0.58#&
T4	0.82 ± 0.60	1.08 ± 0.63#	1.42 ± 0.57#&
T5	0.74 ± 0.49	0.76 ± 0.52	1.20 ± 0.53#&
T6	0.66 ± 0.52	0.70 ± 0.46	1.10 ± 0.54#&

#* P* < 0.05 vs. Group A at the same time point; &* P* < 0.05 vs. Group B at the same time point. *Group A* 0.075% ropivacaine + 0.5 μg/ml sufentanil, *Group B* 0.10% ropivacaine + 0.5 μg/ml sufentanil, *Group C* 0.125% ropivacaine + 0.5 μg/ml sufentanil. *T1* 10 min after analgesia, *T2* 30 min after analgesia, *T3* 1 h after analgesia, *T4* 2 h after analgesia, *T5* the cervix full-dilated time, *T6* the time of fetal delivery

### Analysis of maternal delivery and neonatal status

No significant difference was witnessed in the time between the first stage of labor and the third stage of labor among the three groups (*P* > 0.05). The duration of the second stage of labor in group B (0.10% ropivacaine + 0.5 μg/ml sufentanil) and group C (0.125% ropivacaine + 0.5 μg/ml sufentanil) was longer than that in group A (0.075% ropivacaine + 0.5 μg/ml sufentanil), which was further longer in group C (0.125% ropivacaine + 0.5 μg/ml sufentanil) compared with group B (0.10% ropivacaine + 0.5 μg/ml sufentanil) (*P* < 0.05). However, the second stage of labor in the three groups was within the normal range. Decreased diastolic and systolic blood pressures and increased heart rate were observed in puerperae in group B (0.10% ropivacaine + 0.5 μg/ml sufentanil) in comparison to the other two groups (all *P* < 0.05), and these three indicators in group B women were closer to the normal range. No significant change was found in diastolic blood pressure, systolic blood pressure, and heart rate in group A (0.075% ropivacaine + 0.5 μg/ml sufentanil) and group C (0.125% ropivacaine + 0.5 μg/ml sufentanil) (all *P* > 0.05). The results are detailed in Table 6.

**Table 6:** Comparison of maternal delivery among puerperae in each group ($\overline{x }$ ± SD)

Item	Group A (*n* = 50)	Group B (*n* = 50)	Group C (*n* = 50)
First stage (min)	529.98 ± 89.63	526.95 ± 90.78	532.69 ± 87.68
Second stage(min)	46.40 ± 20.81	57.25 ± 19.16#	69.42 ± 30.07&
Third stage (min)	9.96 ± 2.89	9.85 ± 3.43	10.04 ± 1.99
Systolic pressure (mmHg)	153.86 ± 9.35	125.36 ± 8.53#	155.80 ± 9.22&
Diastolic pressure (mmHg)	106.88 ± 10.70	81.70 ± 8.75#	110.48 ± 11.83&
Heart rate (time/min)	74.46 ± 6.37	80.20 ± 5.32#	76.46 ± 8.09&

#* P* < 0.05 vs. Group A; &* P* < 0.05 vs. Group B. *Group A*, 0.075% ropivacaine + 0.5 μg/ml sufentanil; *Group B*, 0.10% ropivacaine + 0.5 μg/ml sufentanil; *Group C*, 0.125% ropivacaine + 0.5 μg/ml sufentanil

Analysis of the neonatal status indicated that there was no change in PCO_2_, PO_2_, pH, 1 min Apgar score, and 5 min Apgar score among the three groups (all *P* > 0.05) (Table 7).

**Table 7:** Neonatal status analysis in three groups ($\overline{x }$ ± SD)

Item	Group A (*n* = 50)	Group B (*n* = 50)	Group C (*n* = 50)	*P* value
PCO_2_ (mmHg)	52.70 ± 5.74	51.42 ± 5.00	51.72 ± 4.65	0.432
PO_2_ (mmHg)	28.58 ± 4.24	30.26 ± 4.69	29.28 ± 3.85	0.146
pH	7.36 ± 0.13	7.41 ± 0.18	7.39 ± 0.15	0.270
1 min Apgar score	9.62 ± 0.53	9.58 ± 0.50	9.68 ± 0.47	0.604
5 min Apgar score	9.64 ± 0.48	9.72 ± 0.45	9.76 ± 0.43	0.406

*Group A*, 0.075% ropivacaine + 0.5 μg/ml sufentanil; *Group B*, 0.10% ropivacaine + 0.5 μg/ml sufentanil; *Group C*, 0.125% ropivacaine + 0.5 μg/ml sufentanil

### Adverse reactions

According to the results of Table 8, the incidence of adverse reactions of puerperae was compared among the three groups (*P* < 0.05). The incidence of adverse effects in group C (0.125% ropivacaine + 0.5 μg/ml sufentanil) was higher than that in group A (0.075% ropivacaine + 0.5 μg/ml sufentanil) and group B (0.10% ropivacaine + 0.5 μg/ml sufentanil). Nevertheless, there was no change in the incidence of adverse effects between group A (0.075% ropivacaine + 0.5 μg/ml sufentanil) and group B (0.10% ropivacaine + 0.5 μg/ml sufentanil) (*P* > 0.05).

**Table 8:** Comparison of adverse reactions among puerperae in each group (*n*/%)

Adverse reaction	Group A (*n* = 50)	Group B (*n* = 50)	Group C (*n* = 50)
Hypotension	0(0.00%)	1(2.00%)	3(6.00%)
Skin itching	1(2.00%)	2(4.00%)	4(8.00%)
Nausea and vomiting	2(4.00%)	2(4.00%)	3(6.00%)
Urinary retention	2(4.00%)	3(6.00%)	6(12.00%)
Fever	1(2.00%)	1(2.00%)	3(6.00%)
Incidence of adverse reaction	6(12.00%)	9(18.00%)	19(38.00%)#&
*P* value	0.005		

#* P* < 0.05 vs. Group A; &* P* < 0.05 vs. Group B. *Group A*, 0.075% ropivacaine + 0.5 μg/ml sufentanil; *Group B*, 0.10% ropivacaine + 0.5 μg/ml sufentanil; *Group C*, 0.125% ropivacaine + 0.5 μg/ml sufentanil

## Discussion

Epidural blockade is an effective way for labor analgesia, which is beneficial for painless delivery. However, epidural labor analgesia also has some disadvantages, including hypotension, motor blockade, as well as a prolonged second stage of labor (Li et al. 2020). The ideal method for labor analgesia should have a good analgesic effect, improve subject satisfaction, and reduce adverse pregnancy outcomes without influencing the progress of labor (Wang Y and Xu 2020). In clinical anesthesia, a lower concentration of local anesthetic is less likely to develop adverse reactions, but the drug action duration is relatively shorter at the same dosage (Kampe et al. 2004). Therefore, seeking a suitable anesthetic concentration is vital in clinical application. In this work, we aimed to analyze the clinical effect of different concentrations of ropivacaine in the labor analgesia of DPE technique for obese puerperae, and found that the application of 0.1% ropivacaine combined with 0.5 μg/ml sufentanil through the DPE technique has better labor analgesia and fewer adverse reactions.

Ropivacaine is widely used in obstetric anesthesia due to its good analgesic properties without resulting in motor blockade or systemic toxicity (Ahirwar et al. 2014; Wang X et al. 2015). Epidural opioids are also recommended to combine with local anesthetics to decrease the local anesthetic doses and to provide a superior analgesic effect (Ngan Kee et al. 2014). As previously reported, administration of both ropivacaine and sufentanil, as a common approach for labor analgesia, exerts significant effects on postoperative pain management (Carey et al. 2018; Fassio et al. 2018; Katakura et al. 2021). Following the previous publications, 0.1–0.2% ropivacaine is the optimal concentration for epidural labor analgesia (Patkar et al. 2015; Bullingham et al. 2018). As reported, lowering the ropivacaine concentration from 0.2 to 0.125% diminishes the motor block intensity while not influencing the labor duration, cesarean section rate, or instrumental delivery (Sia et al. 1999). Similarly, in this work, we divided the obese puerperae into three groups: group A: 0.075% ropivacaine + 0.5 μg/ml sufentanil; group B: 0.10% ropivacaine + 0.5 μg/ml sufentanil; group C: 0.125% ropivacaine + 0.5 μg/ml sufentanil. The analgesia effect, maternal delivery and neonatal status, and adverse reactions were observed and recorded.

We first found that the analgesia onset time, PCEA pressing time, and ropivacaine consumption in group C were lower and the analgesia time and the first PCEA time were longer than those in group A and group B. At T1–T3 and T5 time points, the VAS scores of group A were higher than those in group B and group C, the Ramsay score of group A was lower than that of group B and group C at time points T2–T3, and the Bromage score of group C at any time point was higher than the other two groups, which suggested that 0.075%, 0.1%, and 0.125% concentrations of ropivacaine compounded with 0.5 μg/ml sufentanil have some analgesic effects during labor and delivery of obese puerperae, of which 0.125% ropivacaine has the best analgesic effect, followed by 0.1% ropivacaine. We also observed that the effect of 0.075%, 0.1%, and 0.125% concentrations of ropivacaine plus 0.5 μg/ml sufentanil on the second course of labor time in obese women was prolonged with increasing concentrations. In addition, we found that the blood pressure and heart rate of puerperae in group B were closer to normal values, suggesting that the 0.1% concentration of ropivacaine had the most effect on maternal blood pressure and heart rate. In the meantime, three different concentrations of ropivacaine had no significant effect on the umbilical artery blood gas analysis indices and Apgar scores at 1st minute and 5th minute in neonates, revealing that 0.075%, 0.1%, and 0.125% concentrations of ropivacaine plus 0.5 μg/ml sufentanil had no obvious effect on neonates during labor analgesia. Furthermore, the incidence of maternal adverse reactions in group C was lower than those in group A and group B, demonstrating that the administration of 0.125% ropivacaine + 0.5 μg/ml sufentanil showed the fewest adverse reactions.

DPE technique with 25-G spinal needles has been revealed to present greater sacral spread, faster analgesia onset and sacral coverage, lower incidence of asymmetric block, as well as lesser requirement of epidural top-up (Lin et al. 2023). Compared with conventional epidural analgesia, DPE analgesia achieves satisfactory pain control by shortening the time, which is beneficial in relieving labor pain. Besides, DPE analgesia is irrelevant to elevated adverse maternal/fetal events (Song et al. 2021; Yin et al. 2022). Shoko Okahara et al. have supported that DPE might be a safer approach to neuraxial analgesia than combined spinal-epidural analgesia in nulliparous women in the early stage of labor (Okahara et al. 2023). Similar to our findings, evidence has shown that epidural anesthesia with 0.075% ropivacaine plus sufentanil can get a good analgesic effect and a shorter second stage of labor (Boulier et al. 2009; Yue et al. 2013). Zhang et al. have stated that women with opioid analgesia show a prolonged labor duration: 0.1% ropivacaine + 0.5 µg/ml sufentanil shows a longer duration of the first stage of labor than the application of 0.167% ropivacaine but did not have an additional impact on either maternal or neonatal outcomes (Zhang L et al. 2021). Also, this research demonstrated that 0.125% ropivacaine had the best analgesia, but 0.1% ropivacaine plus 0.5 μg/ml sufentanil had better labor analgesia in obese women with fewer adverse effects (Ahirwar et al. 2014). Another research has revealed that the effect of 0.075% ropivacaine plus 0.5 mg/ml sufentanil injection through the DPE technique on labor analgesia is shorter than that with 0.1% ropivacaine. Besides, it can reduce the analgesia time, the second stage of labor, diminish the intrapartum febrile rate and result in inflammation (Zhou et al. 2019). All these findings are partly consistent with the results of our work.

In summary, our article underlines that the application of 0.1% ropivacaine combined with 0.5 μg/ml sufentanil through the DPE technique has better labor analgesia and fewer adverse reactions in comparison to 0.075% ropivacaine and 0.125% ropivacaine on labor analgesia in obese puerperae. Nevertheless, labor itself is a complicated process and is influenced by multiple factors. Besides, we did not perform the sample size calculation, which could also be considered as a study limitation. Therefore, the application mode and dosage of labor analgesia need to be further explored in clinical application.

### Supplementary Information

**Additional file 1.**

**Additional file 2.**

**Additional file 3.**

**Additional file 4.**

**Additional file 5.**

**Additional file 6.**

**Additional file 7.**

**Additional file 8.**

## Data Availability

The original contributions presented in the study are included in the article/supplementary materials, further inquiries can be directed to the corresponding author.
